# Incidence and predictors of loss to follow-up among pregnant and lactating women in the Option B+ PMTCT program in Northwestern Ethiopia: a seven-year retrospective cohort study

**DOI:** 10.3389/fgwh.2023.1128988

**Published:** 2023-07-17

**Authors:** Melkalem Mamuye Azanaw, Adhanom Gebreegziabher Baraki, Melaku Kindie Yenit

**Affiliations:** ^1^ Department of Public Health, College of Health Sciences, Debre Tabor University, Debre Tabor, Ethiopia; ^2^ Department of Epidemiology and Biostatistics, Institute of Public Health, College of Medicine and Health Science, University of Gondar, Gondar, Ethiopia

**Keywords:** HIV, women, loss to follow-up, option B+, PMTCT, Northwest Ethiopia

## Abstract

**Introduction:**

Although Ethiopia has implemented the Option B+ program over the past 7 years, loss to follow-up among HIV-positive women remains a major problem for antiretroviral therapy (ART) treatment. This study was conducted to investigate the number of women who dropped out of follow-up after the Option B+ program.

**Methods:**

A retrospective follow-up study was conducted among 403 pregnant and lactating women between June 2013 and December 2019 at health facilities in Northwest Ethiopia. The Cox proportional hazards regression model was used to identify predictors of loss to follow-up. The results were reported as hazard ratios with 95% confidence intervals (CIs) at a significance level of *p* = 0.05.

**Results:**

The overall incidence rate of loss to follow-up was 9.4 per 1,000 person-months of observation (95% CI: 7.40–11.90). According to the multivariable Cox regression, rural residency [adjusted hazard ratio (AHR): 2.30; 95% CI: 1.08–4.88], being a Muslim religion follower (AHR: 2.44; 95% CI: 1.23–4.81), having no baseline viral load measurement (AHR: 4.21; 95% CI: 2.23–7.96), being on ART before enrolment (AHR: 0.30; 95% CI: 0.15–0.62), having drug side effects (AHR:1.82; 95% CI: 1.01–3.33), same-day ART initiation (AHR: 3.23; 95% CI: 1.53–6.84), and having suboptimal adherence level (AHR: 3.96; 95% CI: 2.18–7.19) were significant predictors of loss to follow-up.

**Conclusion:**

The incidence of loss to follow-up is lower as compared to evidence from most African countries but slightly higher than the WHO target. It is better to strengthen and expand viral load measurements for all women and to pay attention to women residing in rural areas with fair or poor adherence levels.

## Introduction

Globally, an estimated 180,000 new pediatric infections were reported annually in 2018 (1). Mother-to-child transmission (MTCT) accounts for 90% of these new pediatric infections and may be transmitted *in utero*, labor, delivery, or breastfeeding (2). Without any intervention, MTCT ranged from 15% to 45%. However, antiretroviral treatment and other interventions can reduce this risk to below 5% in breastfeeding women and 2% in non-breastfeeding women (2–4).

The Joint United Nations Program on HIV/AIDS set a target for member states to have virtual elimination of MTCT to less than 5% and 90% reduction of new human immunodeficiency virus (HIV) infections among young children by 2015, and moved the global commitment to eliminate MTCT by 2020 and the HIV epidemic in 2030 (1). Moreover, the World Health Organization recommends the Option B+ program to prevent MTCT transmission of HIV infection. Option B+ program is the provision of universal, lifelong antiretroviral therapy (ART) for all HIV-infected individuals regardless of CD4 count and WHO clinical staging as a “Test and Treat” approach in 2016 (2, 5–7). These strategies are directly related to an increase in the coverage of antiretroviral medicines taken by pregnant women living with HIV from 51% in 2010 to 80% in 2017 to prevent MTCT (1). In addition to this, these programs contributed to the reduction of maternal mortality by 44% between 1990 and 2015 (8) and the aversion to 1.4 million new child infections since 2010 (1). Despite these efforts, loss to follow-up (LTFU) and poor adherence to drugs remain major challenges in achieving virtual elimination of MTCT of HIV, especially in sub-Saharan Africa (9). Loss to follow-up in the prevention of mother to child transmission (PMTCT) program had an impact on women's access to HIV care and treatment, which led to an advanced HIV stage, increased maternal HIV/acquired immunodeficiency syndrome (AIDS)-related morbidity and mortality, vertical transmission of HIV to newborns, development of drug resistance, and missed opportunities for family planning (2, 10). Moreover, LTFU has consequences for future pregnancies of child health and survival and increases the risk of transmission if the partner is serodiscordant (2).

Different studies have indicated that the incidence of LTFU among HIV-positive pregnant and lactating mothers in the PMTCT program varies in different countries. The incidence rates of LTFU in Brazil (11) and Myanmar (12) among mother–child pairs and pregnant women in the PMTCT program were 15.4% and 7 per 1,000 person-years, respectively. Moreover, the incidence of LTFU among pregnant and lactating mothers under the Option B+ program remains high in different African countries, ranging from 16% to 53.7% (9, 13–26). Previous studies in Ethiopia have shown that the rate of LTFU among women under the Option B+ program varies from region to region. A retrospective follow-up study at different public health facilities in the northeast regions of Ethiopia showed that the cumulative incidence of LTFU was 16.5% (27). Other findings revealed that cumulative incidence was 15.4% with an incidence rate of 9 per 1,000 person-months (PM) in western Ethiopia (28) and 18.2% in western Oromia (29).

Based on different studies, maternal age less than 25 years (9, 13, 15, 21, 26, 30, 31), lower educational status (15, 32), unmarried marital status (19), unemployment status (26), positive partner HIV status (9, 16), baseline low CD4 cell counts and high viral load measurement (33, 35), WHO clinical stage 3/4 (23), treatment on the same day of initiation as HIV diagnosis (20, 23), new enrolment in PMTCT services, drug side effects, and changes in the treatment regimen (15, 36–38) were significant predictors of LTFU.

Although there are studies on LTFU among the general adult population on ART care, there are no published studies on the incidence of LTFU and its predictors among HIV-positive pregnant and lactating mothers on ART treatment since the start of the Option B+ program. The findings of this study will help health institution managers and programmers develop evidence-based interventions to promote retention in care for both mothers and infants, because early identification of magnitude and factors helps identify vital sites and improve women with HIV by enhancing viral suppression. The findings of this study will also assist policymakers and programmers in focusing on the major identified risk factors for LTFU among HIV-positive women to improve PMTCT services and eradicate HIV as planned in 2030.

## Methods

### Study setting

This study was conducted at health facilities in Gondar, Northwest Ethiopia. This town is located in the Amhara region 748 km from Addis Ababa. It has one referral hospital that provides services for over 5 million people in the catchment area (39). The PMTCT program began on June 23, 2013, and since then 1,049 women with HIV were enrolled for PMTCT services and treatment through December 31, 2019.

### Study design and population

This institution-based retrospective follow-up study was conducted among pregnant and lactating women in the Option B+ PMTCT program. Data were extracted from February to April 2020. Pregnant and lactating women in the PMTCT program from June 23, 2013, to December 31, 2019, were included in the study. The study included only pregnant and breastfeeding women who were enrolled in long-lasting ART PMTCT services for at least 3 months before the end of data collection. We excluded patients with incomplete PMTCT record regarding maternal cohort outcomes and data inconsistencies regarding ART confirmation and initiation in the Option B+ program. A total of 403 pregnant and breastfeeding women's charts were reviewed after selection using simple random sampling from the PMTCT register based on the inclusion criteria.

### Study variables and definitions

The outcome variable in this study was the incidence of LTFU in the Option B+ PMTCT program.

#### Operational definitions

**LTFU** is defined as missing 90 days after the last documented visit as per recently developed simplified tools to measure retention in care in ART programs (40, 41). **Event:** LTFU, which is 3 months after the last documented visit under Option B+ PMTCT and not recorded as “dead,” “retained,” or “transferred-out” on the patient's PMTCT logbook or medical cards (4). **Censored:** A patient did not develop an event or LTFU that could be death, transferred-out, treatment completed, or receiving treatment when the study ended.

**Time to LTFU:** Time in months from the beginning of treatment under the Option B+ PMTCT program to LTFU under the program. **Recent adherence level:** Adherence was classified as good, fair, or poor, according to the percentage of drug dosage calculated from the monthly total dose of ART drugs. Describe as **good** (equal to or greater than 95% or ≤missing less than or equal to 2 out of 30 doses or missing 3 or less from the 60 doses), **fair [(**85%–94%) adherence or missing 3–5 doses out of 30 tabs or 3–9 tablets from 60 doses], or **poor** (less than 85% or missing ≥6 tablets out of 30 tabs or >9 tabs from 60 tabs) (42). The functional status of women was defined based on the WHO criteria as follows: **Working** is defined as the ability to perform usual work inside or outside the home. **Ambulatory**: able to perform activities of daily living. **Bedridden**: inability to perform activities of daily living (43).

### Data collection procedure and quality assurance

The data sources included the Federal Ministry of Health patient card, ART intake forms, HIV care follow-up, and the PMTCT register using a data extraction checklist. The components of the checklist are sociodemographic characteristics, immunologic, treatment, clinical, behavioral, follow-up, and outcome variables. Records of patients in the last 7 years after the Option B+ program were extracted based on the inclusion criteria. Six data collectors and two supervisors were recruited to extract data from the PMTCT service records at the University of Gondar Comprehensive and Specialized Hospital. Data reviewers were trained with BSc midwives working in the PMTCT clinic. Data were extracted using a structured extraction tool prepared for the study. The data extraction checklist was prepared based on the information contained within the patient registration and follow-up cards, according to national guidelines.

### Statistical analysis

After collecting the data, they were cleansed, coded, and entered into Epi Data version 4.6.0.0. They were then exported to STATA version 14 (StataCorp, College Station, TX, United States) for further analysis. The incidence rate was computed using person-month observations by adding the amount of time spent by study participants during the follow-up period. Kaplan–Meier non-parametric survival analyses were performed to estimate the cumulative survival probability of LTFU at a specific time after the PMTCT program and the Nelson–Aalen method was used to generate a cumulative hazard function. Univariate Cox proportional hazards were fitted to the predictors of LTFU among women in the Option B+ PMTCT program for complete data. Potential predictors signiﬁcantly associated with LTFU in the univariate models (*p* < 0.25) were evaluated using a multivariable model. To identify the combination of factors that best predicted LTFU, the backward stepwise Cox proportional hazards model evaluated the inclusion or exclusion of potential predictors at each step. The model with the highest log likelihood was chosen and checked for individual variation using a univariate frailty model. The proportional hazard assumption and fit of the model were checked using the Schoenfeld global test and Cox-Snell residual plot, respectively. Finally, results were reported as a hazard ratio with 95% CI and examined at a significance level of *p* = 0.05 using a multivariable Cox proportional hazard model.

## Results

### Sociodemographic and maternal-related characteristics

The mean [± standard deviation (SD)] age of the mothers was 27.6 ± 4.7 years. Overall, 98 (24.3%) women were aged 15–24 years old. The majority of study participants 248 (61.5%) were urban dwellers. A total of 186 participants (46.1%) were housewives. Of the 403 observations, 359 (89.1%) women were pregnant during ART treatment and 169 (41.9%) were newly enrolled in ART treatment. Approximately 70 (17.4%) women had a mid-upper arm circumference (MUAC) (<23 cm) level. Overall, 222 (55.1%) of the partners were positive for HIV (Table 1).

**Table 1:** Baseline sociodemographic, maternal-related characteristics of pregnant and breastfeeding women on Option B+ from June 2013 to December 2019 in Northwest Ethiopia (*n* = 403).

Variables	Category	Frequency (41)
Age in years	Mean ± SD	27.6 ± 4.7
	15–24	98 (24.3)
	25–29	160 (39.7)
	30–45	145 (36.0)
Place of residence	Urban	248 (61.5)
	Rural	155 (38.5)
Marital status	Single	53 (13.2)
	Married	264 (65.5)
	Widowed	33 (8.1)
	Divorced	53 (13.2)
Religion	Orthodox Christian	328 (81.4)
	Muslim	49 (12.2)
	Others (protestant, catholic)	26 (6.4)
Educational status	No education	126 (31.3)
	Primary	112 (27.8)
	Secondary	110 (27.3)
	Above secondary	55 (13.6)
Occupational status	Housewife	186 (46.1)
	Governmental employee	62 (15.4)
	No-governmental employee	23 (5.7)
	Daily laborer	99 (24.6)
	Others^a^	33 (8.2)
Number of pregnancies	One	147 (36.5)
	Multiple	256 (63.5)
Enrolment Status of women	Pregnancy	359 (89.1)
	Breastfeeding	44 (10.9)
Enrolment type to PMTCT	New	169 (41.9)
	On ART before enrolment	234 (58.1)
Recent MUAC level	≥23 cm	333 (82.6)
	<23 cm	70 (17.4)
Partner HIV status	Positive	222 (55.1)
	Negative	131 (32.5)
	Unknown/not done	50 (12.4)
Disclosure status	Yes	315 (78.2)
	No	88 (21.8)
Total		403 (100.0%)

^a^ Others: student and merchant.

### Clinical, laboratory, and treatment-related characteristics

The mean ± SD of CD4 was 419.1 ± 224.0 cells/ml^3^ with a CD4 count greater than 350 cells/mm^3^ of 238 (59.1%) at baseline. Of the total study participants, 344 (85.4%) were classified as WHO clinical stage one followed by WHO clinical stage two 37(9.2). The combination therapy *AZT-3TC-EFV* was the second predominant regimen prescribed during enrolment for 105 (26.1%) women, followed by *TDF-3TC-EFV* for 239 (59.3%). Overall, 132 (32.8%) women were anemic during enrolment. The majority (83.6%) of the women had good adherence levels, and 72 (17.9%) developed ART treatment-related side effects during PMTCT enrolment. Overall, 279 (69.9%) women had at least one recorded viral load measurement within 3 months of PMTCT enrolment (Table 2).

**Table 2:** Clinical, laboratory, and treatment-related characteristics of pregnant and breastfeeding women on Option B+ from June 2013 to December 2019, Northwest Ethiopia.

Variables	Category	Frequency (41)
TB screening status	Negative	379 (94.0)
	Positive	24 (6.0)
INH prophylaxis	No	351 (87.1)
	Yes	52 (12.9)
Comorbidity	Yes	27 (6.7)
	No	376 (93.3)
Initial ART regimen	*AZT-3TC-NVP*	105 (26.1)
	*AZT-3TC-EFV*	29 (7.2)
	*TDF-3TC-EFV*	239 (59.3)
	*TDF-FTC-NVP*	22 (5.4)
	Others	8 (2.0)
Change of ART regimen	No	344 (85.4)
	Yes	59 (14.6)
Anemia status	Not anemic	271 (67.2)
	Anemic	132 (32.8)
Adherence level	Good	337 (83.6)
	Fair/Poor	66 (16.4)
Maternal CPT	No	223 (53.3)
	Yes	180 (44.7)
Drug side effects	No	331 (82.1)
	Yes	72 (17.9)
WHO clinical stage	Early stage (I/II)	381 (94.6)
	Late stage (III/IV)	22 (5.4)
Functional status	Working	355 (88.1)
	Ambulatory/bedridden	48 (11.9)
Time of ART initiation	Later	144 (35.7)
	Same day	259 (64.3)
CD4 cell count (cells/mm^3^)	Mean ± SD	419.1 ± 224.0
	Less than 200	60 (14.9)
	200–350	105 (26.1)
	Greater than 350	238 (59.1)
Baseline viral load taken	Yes	279 (69.2)
	No	124 (30.8)
Total		403 (100.0%)

CPT, co-trimoxazole preventive therapy; AZT, Zidovudine; 3TC, Lamivudine = Nevirapine; EFV, Efavirenz = Tenofovir Disoproxil Fumarate; FTC, Emtricitabine; Others, 1 g (ABC + 3TC + NVP), 2f (AZT-3TC-ATV/r), second-line treatment; TB, Tuberculosis; INH, Isoniazid; Comorbidity, heart disease and/or renal diseases and/or hypertension, and/or diabetes mellitus.

### Maternal PMTCT cohort outcome of the study

#### Survival status of study participants

**Figure 1:**
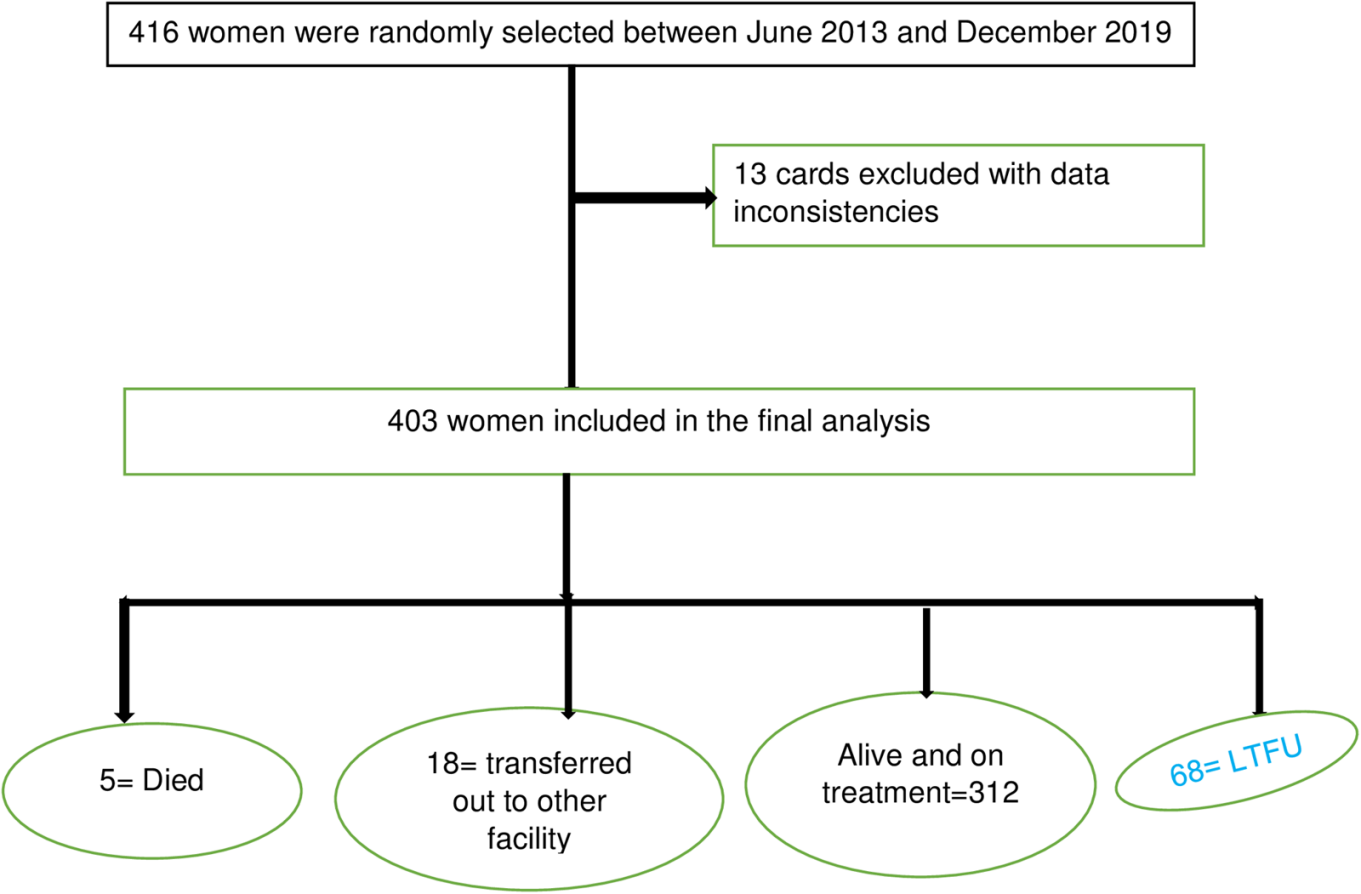
Consort diagram with total number of eligible patients and final number of women included in the analysis.

Overall, 6.8% (95% CI: 13.5–20.8) of women were LTFU to ART treatment by completing the PMTCT program, and five (1.2%) women died during ART treatment of PMTCT. About 403 study participants were followed for a mean ± SD time of 17.9 (±7.2) months. Of the total observations, 12 (2.9%), 31 (7.7%), 53 (13.2%), and 68 (16.8%) women were LTFU at the end of 6, 12, 18, and end months of the PMTCT program, respectively. Three hundred and thirty-five (83.1%) observations were censored (retained, transferred out, or died) at the end of the enrolment (Figure 1).

#### Incidence rate of LTFU

**Figure 2:**
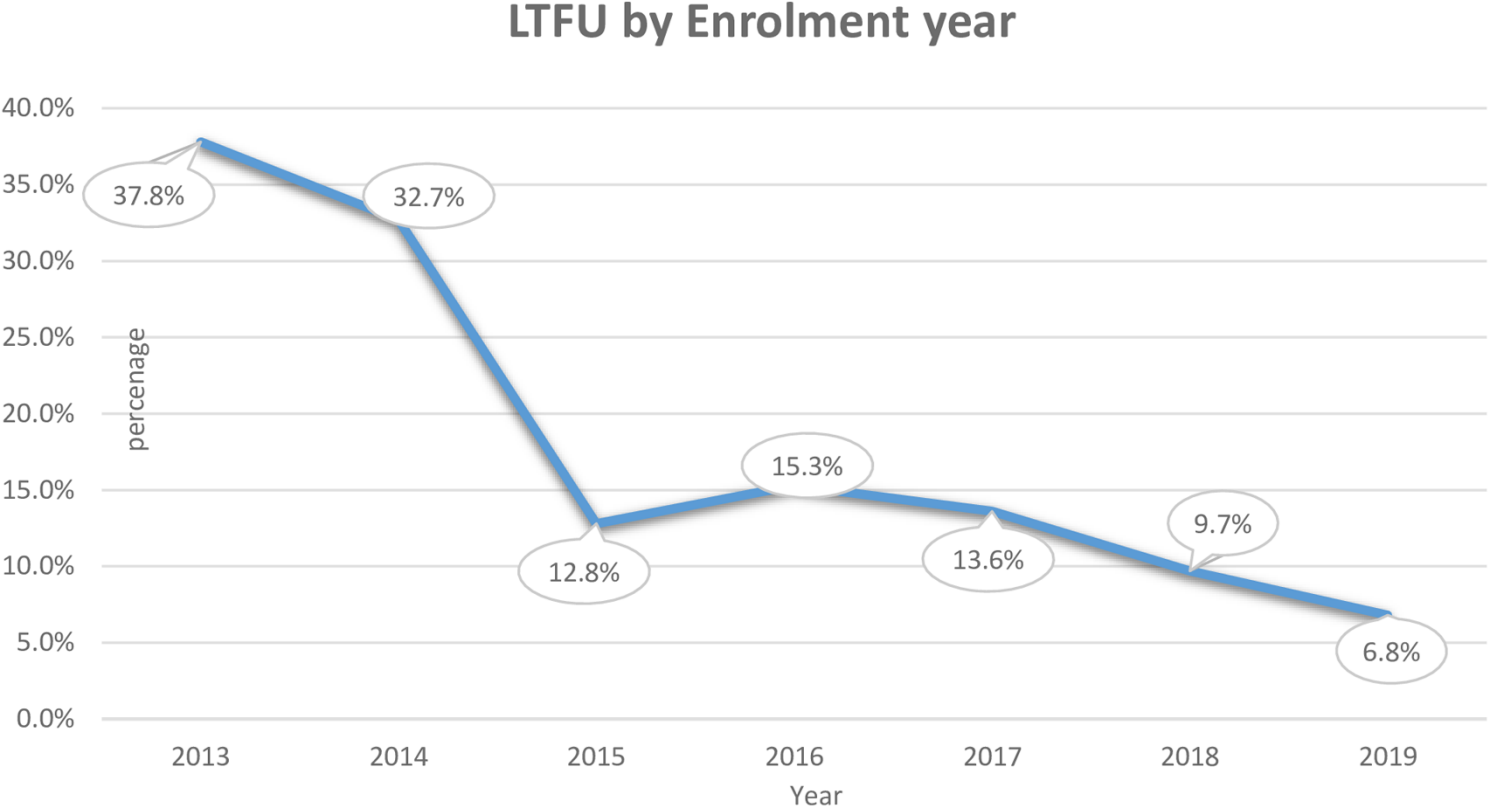
Incidence of LTFU by enrolment year among pregnant and breastfeeding women on Option B+ PMTCT program from June 2013–December 2019 in Northwest Ethiopia.

We calculated the incidence rate by taking the denominator as PM because the study was a dynamic cohort. During the follow-up period, a total of 7,215 person-months’ time risk was observed with an overall incidence rate of LTFU 9.4/1,000 person-months (95% CI: 7.4–11.9) by the end of PMTCT follow-up. The incidence proportion of LTFU was dramatically declining from the initiation of the program in 2013 from 38% to 6.8% in 2019 (Figure 2).

**Figure 3:**
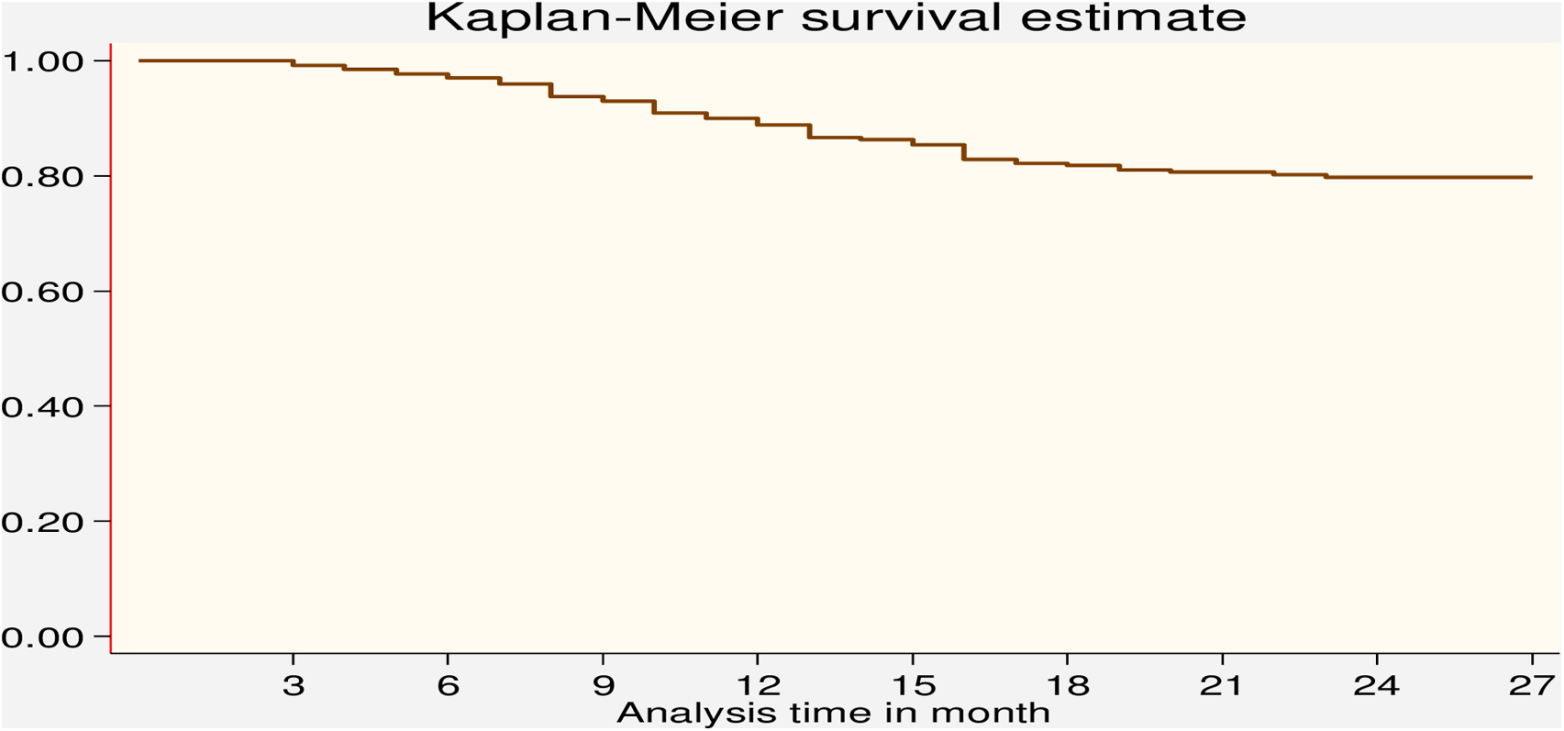
Kaplan–Meier survival estimate of LTFU among pregnant and breastfeeding women on Option B+ PMTCT program from June 2013–December 2019 in Northwest Ethiopia.

**Figure 4:**
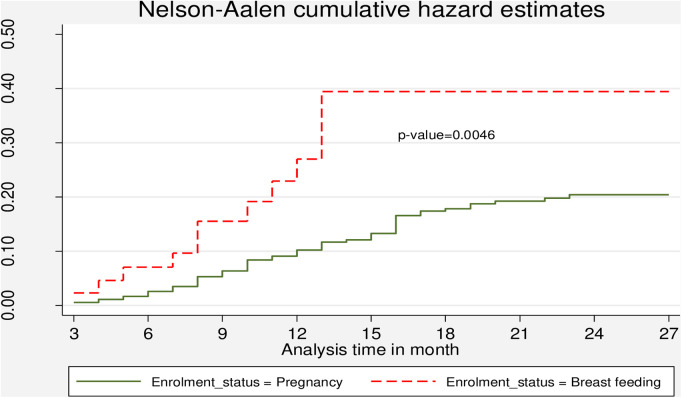
Cumulative hazard estimate of LTFU among pregnant and breastfeeding women on Option B+ PMTCT program by from June 2013–December 2019 in Northwest Ethiopia by enrolment status.

The Kaplan–Meier method for the time to LTFU after ART initiation during the PMTCT follow-up period showed that close to 80% of the participants were still in care after 12 months of follow-up (Figure 3). The cumulative hazard estimate of LTFU showed a difference in the enrolment status of the PMTCT program. The cumulative hazard of LTFU among HIV-positive lactating women was higher than pregnant women during PMTCT enrolment (Figure 4).

#### Incidence of LTFU among different levels of predictor variables

Overall, the incidence rate was higher for women residing in rural areas (19.3 per 1,000 person-months) and lower for mothers residing in urban areas (4.1 per 1,000 person-months). The l-rank test also showed that women had a significant difference in LTFU between the rural and urban areas. Moreover, it shows that the incidence of LTFU was significantly different according to the patient's marital status, religion, status of women during enrolment, enrolment type, MUAC level, anemia status, adherence level, CD4 cell count, ART initiation time, drug side effects, and baseline viral load so that these variables were included in the binary cox regression (Table 3).

**Table 3:** Comparisons of LTFU among different levels of baseline predictor variables using a log-rank test from pregnant and breastfeeding women on Option B+ PMTCT program in Northwest Ethiopia (June 2013–December 2019).

Variables	Category	LTFU		Log-rank test	
		IR/1,000	PMO	*X* ^2^	*p-*value
Age in years	15–24	7.9	1,780		
	25–29	9.2	2,818	0.84	0.6580
	30–45	10.7	2,617		
Place of residence	Urban	4.1	4,675		
	Rural	19.3	2,540	40.98	<0.0001
Marital status	Single	21.5	838		
	Married	7.2	4,866	17.11	0.0002
	Widowed/divorced	9.9	1,511		
Religion	Orthodox Christian	7.8	6,002		
	Muslim	19.5	718	10.93	0.0042
	Others^a^	14.1	495		
Educational status	No education	13.1	2,139		
	Primary	7.3	2,057	4.26	0.2350
	Secondary	8.1	1,985		
	Above	8.7	1,034		
Employment status	Housewife	7.8	3,322		
	Government employee	9.8	1,120	5.74	0.2190
	Non-government employee	19.8	405		
	Daily laborer	9.5	1,789		
	Others^a^	10.4	579		
Number of pregnancies	Single	9.8	2,637		
	Multiple	9.2	4,578	0.11	0.7418
Partner HIV status	Positive	8.6	4,074		
	Negative	7.3	2,326	10.61	0.0050
	Unknown/not done	19.6	815		
Disclosure status	Yes	6.9	5,730		
	No	18.9	1,485	17.49	<0.0001
Enrolment status	Pregnancy	8.4	6,647		
	Breastfeeding	21.1	568	8.05	0.0046
Enrolment type	New	13.7	2,698		
	On ART before enrolment	6.8	4,517	8.44	0.0037
Comorbidity	No	8.0	6,488		
	Yes	22.2	727	13.51	0.0002
MUAC level	≥23 cm	5.6	6,203		
	<23 cm	32.6	1,012	67.16	<0.0001
Anemia status	Not anemic	4.3	5,128		
	Anemic	22.0	2,087	48.56	<0.0001
Maternal CPT	No	4.5	4,017		
	Yes	15.6	3,198	23.81	<0.0001
Initial ART regimen	*AZT-3TC-NVP*	10.3	1,933		
	*AZT-3TC-EFV*	5.2	582		
	*TDF-3TC-EFV*	10.5	4,091	4.58	0.2050
	Other	3.3	609		
WHO clinical stage	Early stage(I/II)	8.5	6,908		
	Late stage (III/IV)	29.3	307	13.06	0.0003
Functional status	Working	7.2	6,538		
	Ambulatory/bedridden	31.0	677	37.27	<0.0001
Adherence level	Good	4.4	6,310		
	Fair/Poor	44.2	905	134.86	<0.0001
CD4 cell count in cells/ml	>350	6.0	4,340		
	200–350	7.8	1,917	14.69	0.0001
	<200	28.2	958		
ART initiation time	Same day	11.5	4,707		
	Latter	5.6	2,508	5.86	0.0155
ART side effects	No	5.1	6,104		
	Yes	33.3	1,111	79.40	<0.0001
Baseline viral load taken	Yes	3.1	5,196		
	No	25.8	2,019	79.00	<0.00001

IR, incidence rate of loss to follow-up; PMO, person-month observation.

Comorbidity included chronic illness, tuberculosis, and opportunistic infections.

^a^ Others: student and merchant.

### Factors affecting loss to follow-up from PMTCT service

Predictors included in the multivariable Cox regression analysis were those with a *p*-value <0.25 in the bivariable analysis, and 21 variables were selected in the first step of model building. After running backward stepwise variable selection and considering multicollinearity, the first group was selected as the best model from the likelihood ratio, which included full (17) variables in the multivariable analysis.

According to the multivariable Cox regression analysis, rural residents were at a 2.30 higher risk of LTFU (AHR: 2.30; 95% CI: 1.08–4.88) as compared to urban residents. Muslim religious followers were at 2.44 times higher risk of LTFU (AHR: 2.44; 95% CI: 1.23–4.81) as compared to Orthodox Christian religious followers. The risk of LTFU for participants who started ART on the same day as HIV diagnosis was 3.23 times more likely than the latter initiation (AHR: 3.23; 95% CI: 1.53–6.84). Participants who were on ART before PMTCT enrolment had a 70% increased risk of LTFU compared with newly enrolled patients (AHR: 0.30; 95% CI: 0.15–0.62). During the last PMTCT follow-up, LTFU among participants who had drug side effects was 82% higher than that among their counterparts (AHR: 1.82; 95% CI: 1.01–3.33). Participants with suboptimal adherence status (fair/poor) had a 3.96 times higher risk of LTFU (AHR: 3.96; 95% CI: 2.18–7.19) than participants with good adherence. Finally, the risk of LTFU among women who had no viral load measurement was 4.21 times higher than those who had viral load measurement during enrolment (AHR: 4.21; 95% CI: 2.23–7.96) (Table 4). The global test results showed *p* > 0.05; thus, we did not violate the proportional assumption.

**Table 4:** Multivariable Cox regression of LTFU among pregnant and breastfeeding women on Option B+ PMTCT program from June 2013–December 2019 in Northwest Ethiopia.

Variables		Censored	LTFU	Crude hazard ratio	Adjusted hazard ratio
				CHR (95% CI)	AHR (95% CI)
Residence	Urban	229	19	1	1
	Rural	106	49	4.75 (2.79–8.07)	2.30 (1.08–4.88) *
Religion	Orthodox	281	47	1	1
	Muslim	35	14	2.52 (1.38–4.57)	2.44 (1.23–4.81)*
	Others^a^	19	7	1.84 (0.83–4.07)	2.33 (0.87–6.21)
Marital status	Married	229	35	1	1
	Single	35	18	3.12 (1.77–5.52)	1.57 (0.76–3.23)
	Divorced/widowed	71	15	1.38 (0.76–2.53)	0.77 (0.36–1.65)
Disclosure status	Yes	275	40	1	1
	No	60	28	2.67 (1.65–4.34)	0.91 (0.47–1.81)
ART initiation time	Latter	130	14	1	1
	Same day	205	54	2.02 (1.12–3.65)	3.23 (1.53–6.84)*
Comorbidity	No	307	52	1	1
	Yes	28	16	2.72 (1.55–4.77)	0.77 (0.37–1.59)
MUAC level	Greater or equal to 23 cm	298	35	1	1
	Less than 23 cm	37	33	5.78 (3.58–9.33)	1.79 (0.91–3.52)
Anemia status	Not anemic	249	22	1	1
	Anemic	86	46	5.04 (3.03–8.39)	1.78 (0.97–3.26)
Clinical stage	Early stage (I/II)	322	59	1	1
	Late stage (III/IV)	13	9	3.35 (1.66–6.78)	0.87 (0.33–2.3
Functional status	Working	308	47	1	1
	Ambulatory/bedridden	27	21	4.33 (2.58–7.26)	1.51 (0.63–3.61)
Maternal CPT	No	205	18	1	1
	Yes	130	50	3.49 (2.03–5.98)	1.84 (0.95–3.58)
Enrolment status	Pregnancy	303	56	1	1
	Breastfeeding	32	12	2.39 (1.28–4.49)	0.97 (0.95–2.25)
Enrolment type	New	132	37	1	1
	On ART before enrolment	203	31	0.50 (0.31–0.81)	0.30 (0.15–0.62)**
Side effects	No	300	31	1	1
	Yes	35	37	6.52 (4.04–10.52)	1.82 (1.01–3.33) *
ART adherence level	Good	309	28	1	1
	Fair/poor	26	40	10.19 (6.26–16.58)	3.96 (2.18–7.19)***
Baseline Viral load taken	Yes	263	16	1	1
	No	72	52	8.29 (4.73–14.52)	4.21 (2.23–7.96)***
Baseline CD4 cell count	<200	33	27	1	1
	200–350	90	15	0.28 (0.15–0.54)	1.19 (0.50–2.83)
	>350	212	26	0.22 (0.13–0.37)	1.53 (0.67–3.49)

^a^ Others: protestant and Catholic religions.

* Significant at *p-*value 0.05.

** Significant at *p-*value 0.01.

*** Significant at *p-*value 0.001.

## Discussion

Loss to follow-up is a major challenge in PMTCT programs, which leads to the advanced stage of HIV, increases maternal HIV/AIDS-related morbidity and mortality, enables the vertical transmission of HIV to newborns, facilitates the development of drug resistance, and missed opportunities for family planning (40). Nationally, there is a target for fulfilling the 90-90-90 strategy as a percentage of currently receiving antiretroviral therapy among all adults and children living with HIV to be 90%, which is to decrease LTFU to less than 10% (5). Therefore, this retrospective record review was conducted to determine the incidence and predictors of LTFU among pregnant and lactating women in the Option B+ PMTCT program in Northwest Ethiopia.

The overall incidence density of LTFU in the current study was 9.4 per 1,000 person-months by the end of the PMTCT follow-up period This finding is in agreement with a previous study conducted in Nekemte Hospital, Western Ethiopia (9 per 1,000 person-months observations) (28). This is due to the similarity in study times at which nationally different strategies were adopted to increase ART coverage and adherence. Among these, ART drug refill and clinical follow-up, including laboratory investigations, were performed in advance at the ART/PMTCT clinic. In addition, case managers who are trained lay workers, most of whom are people living with human immune deficiency virus, provide adherence and psychosocial services at these ART health facilities to decrease interruption from services (44, 45). However, this finding is lower than that reported in study from Northeast Ethiopia (14.8 per 1,000 person-months observations) (27). This difference might be due to the difference in the study time that the study was initiated. The current study includes recent year data at which most strategies at a country level were implemented to decrease LTFU, such as bringing services closer to communities by expanding ART sites to above 1,500 health facilities and increasing service provision by expanding trained health personnel to decrease waiting times at the facility compared to previous studies. The variation could also be explained by the difference in the study settings, since this study was conducted at 1 referral hospital whereas a previous study was conducted in 4 hospitals and 10 health centers. Studies have shown that the magnitude of loss to follow-up varies according to the level of health institution (23, 27). Moreover, the lower incidence of LTFU in the current study might be because different programs and measures have been implemented in the country in recent years to decrease the rate of LTFU women with HIV infection. Loss to follow up will be decreased by increasing trained human power, including midwives, frequent follow-up schedules, and better drug preparation (fixed-dose ART treatment) by providing better consideration for mothers to implement the program effectively (40). It is also lower than those of studies conducted in different African countries such as Uganda (15, 16, 18), South Africa (24), Malawi (14, 46), and Kenya (19). This discrepancy might be due to the differences in study time, operational definition of the outcome variable, and characteristics of the study participants. For example, the study period in Malawi was a 3-year record review (14), whereas the current study incorporated recent years’ data that had improved ART coverage. Another explanation for the discrepancy in incidence rate was the characteristics of study participants; the study in Uganda incorporated 92% of the population with a rural place of residence (16) compared to only 38.5% in the current study. The operational definitions of studies in Kenya and South Africa (26, 32) were missing 6 months until the last follow-up visit compared to 3 months in the current study. Furthermore, the rate of LTFU in the current study was lower than that in a study conducted in Myanmar, which was 7 per 1,000 person-years (11). This difference might be because the study in Myanmar included only pregnant women. Pregnancy-related symptoms and signs during antenatal care clinics (17) have a chance of dropping out from the PMTCT clinic (47).

The current study showed that the risk of LTFU among women residing in rural areas was higher than that among women residing in urban areas. Supporting findings have been reported in previous studies in Ethiopia and Brazil (10, 28, 48). Possible explanations for this might be that mothers from remote areas are forced to travel long distances to get to the nearest hospital, which necessarily involves high costs that lead to LTFU (16), and cannot easily get transport services due to poor/lack of road construction, making women walk long distances barefoot, which leads them to be less likely to adhere to Option B+ strategy (29), resulting in missing appointments (15, 31). This justification is also supported by the report that lack of access to healthcare services leads to poor adherence and LTFU to Option B+ PMTCT drugs (15). Although this was not assessed in the current study, an additional explanation for the high risk of LTFU is that, in a rural setting, transport is costly because most mothers are farmers and housewives with low socioeconomic status (16).

This study also found that women who had no baseline viral load measurements were more likely to have LTFU than those who had baseline viral load measurement within 3 months of PMTCT enrollment. This finding is supported by a study in Nigeria among the general population, missing viral load measurements increasing LTFU (49). This might be because when viral load measurements were taken during PMTCT enrolment, the healthcare provider classified women as high risk with a viral load of more than 1,000 copies/mm^3^ and low risk with a viral load of less than 1,000 copies/mm^3^. Therefore, those in high-risk categories will be followed carefully and frequently in order not to miss the appointment time since it is a gold criterion to know the women in a good way or adhere to service provisions (40). Another possible explanation for the high rate of LTFU in those who had no baseline viral load measurement is that viral load measurement was implemented in advance after 2016 in Ethiopia. Another possible explanation could be that taking viral load measurement at baseline raises women's HIV-related literacy and awareness and might engage women in care (45).

Moreover, women with fair/poor drug adherence were more likely to have LTFU than women with good adherence. This finding is supported by studies conducted in Ethiopia (48) and Malawi (22). This might be because poor adherence to drugs is due to the feared side effects resulting in stopping taking ART treatments and lack of knowledge toward the importance of adherence to all appointments lead to stopping/missing the schedule of ART treatment (18).

The current study revealed that the risk of LTFU in women who started ART on the same day following HIV diagnosis was higher than that in women who started ART later following HIV diagnosis. This finding agrees with those of studies conducted in Northeast Ethiopia (27) and Malawi (20). This might be due to the need for sufficient time and information for clients to adjust and prepare themselves for lifetime treatment, psychologically, socially, and physically. Moreover, the reason for loss to follow-up for those women who started the same-day initiation might be due to the combined effect of ART side effects at the time of initiation and pregnancy-induced physiological side effects, such as regurgitation, nausea, and vomiting, which leads to loss in treatment follow-up. However, a study in southern Ethiopia suggested that pregnant women who started ART at the time of HIV diagnosis were more likely to adhere to Option B+ ART, resulting in increased retention in HIV care (29), which is contrary to the findings of this study. The current study is also against the study in South Africa, which showed that same-day antiretroviral therapy initiation in pregnancy is not associated with engagement in care (35). This might be due to the difference in study participants, in which the study in South Africa included only pregnant women.

The risk of LTFU among women who were receiving ART before PMTCT enrolment was lower than that among women who were enrolled in PMTCT. This finding is in agreement with studies conducted in different countries in Africa (19, 23, 28). A possible explanation for this might be that a known HIV woman on ART before enrolment had experienced ART treatment and might have good awareness about ART treatment, drug side effects, and drug adherence than a newly enrolled woman. Evidence also showed that a new HIV diagnosis during routine antenatal screening can be attended by different degrees of shock and denial and may lead to difficulty accepting immediate initiation of lifelong treatment, resulting in a loss to follow-up (46). This study also supported a previous study done in South Africa, which stated that being newly diagnosed with HIV was a positively significant predictor of disengagement from ART treatment (24).

The findings of the current study also revealed that women who had ART side effects during PMTCT follow-up had a higher risk of LTFU than those who had no ART side effects. The findings of this study are supported by studies in Uganda (16) and Malawi (9, 26, 36). This might be due to less counseling on the side effects of ART and less support for women experiencing challenges with tolerability, including options to switch regimens (24).

All in all, the incidence rate of LTFU was higher in the last month of the PMTCT follow-up period, implying a lack of proper linkage and referral systems between PMTCT services and ART clinics. In addition, contrary to the current study, variables such as educational status, maternal age, and baseline CD4 cell count showed statistically significant associations with LTFU among HIV-infected women receiving PMTCT services in previous studies conducted in different African countries (15, 23, 24, 26, 27, 36). However, these variables were not statistically significant in this study. This difference might be because the predictors of LTFU varied from one geographical area to another due to the differences in the economic status of the study participants and infrastructure in the health facilities.

### Limitations of the study

This study has some limitations that must be considered before interpreting the results. First, as we conducted a thorough review of records, we did not include important predictors of LTFU such as stigma, distance to hospital, and social support. Second, the true incidence of LTFU might have been underestimated and/or overestimated because of incomplete documentation of data and classification errors. Finally, there are limitations related to the abstraction of routinely collected data.

## Conclusion

The current study revealed that the incidence of LTFU dramatically and consistently decreased as Option B+ matured from 38% in 2013 to 6% in 2019. A high rate of LTFU was observed in the last month of PMTCT follow-up. The study also found a lower rate of LTFU among HIV-positive women than in previous studies, but it was slightly higher than the WHO target. The place of residence, recent adherence level, baseline viral load measurement, known HIV status, time of ART initiation, drug side effects, and religion were found to be predictors of LTFU. We recommend that the hospital pay more attention to rural dwellers to decrease LTFU in collaboration with the Zonal Health Department, the Woreda Health Office, and stakeholders. Information about optimal ART drug adherence and viral load measurement is recommended because these are the main contributing factors to the decline in LTFU over time. Better consideration should be given to ART side effects that are in adherence and retention. We have recommended research on primary data that incorporates maternal conditions, community, behavioral, and cultural factors such as women's attitudes toward maternal ART treatment using qualitative studies. Moreover, it is better to be done as a prospective and prognostic model for the risk prediction of LTFU. The results of this study will help achieve the goal of the Joint United Nations Program on HIV/AIDS, which is to reduce mother-to-child transmission of HIV by 5%.

## Data Availability

The raw data supporting the conclusions of this article will be made available by the authors, without undue reservation.

## References

[1] Vrazo AC Sullivan D Ryan Phelps B. Eliminating mother-to-child transmission of HIV by 2030: 5 strategies to ensure continued progress. J Glob Health Sci Pract. (2018) 6(2):249–56. 10.9745/GHSP-D-17-00097 PMC6024627 29959270

[2] World Health Organization (WHO). Consolidated guidelines on the use of antiretroviral drugs for treating and preventing HIV infection: Recommendations for a public health approach. 2nd ed. Geneva: World Health Organization (2016). 27466667

[3] Joint United Nations Programme on HIV/AIDS. *Start free stay free AIDS free*. Progress report. Geneva, Switzerland (2019). Available at: https://www.unaids.org/en/resources/documents/2019/20190722_UNAIDS_SFSFAF_2019

[4] https://www.unaids.org/en/resources/fact-sheet.

[5] World Health Organization (WHO). Programmatic update use of antiretroviral drugs for treating pregnant women and preventing HIV infection in infants. Geneva: HIV/AIDs Program (2012).

[6] Options B and B+: key considerations for countries to implement an equity-focused approach. Geneva: UNICEF (2012).

[7] Update WP. Use of antiretroviral drugs for treating pregnant women and preventing HIV infection in infants. Geneva: WHO (2012).

[8] You D Hug L Ejdemyr S Idele P Hogan D Mathers C Global, regional, and national levels and trends in under-5 mortality between 1990 and 2015, with scenario-based projections to 2030: a systematic analysis by the UN inter-agency group for child mortality estimation. J Lancet. (2015) 386(10010):2275–86. 10.1016/S0140-6736(15)00120-8 26361942

[9] Atanga PN Ndetan HT Achidi EA Meriki HD Hoelscher M Kroidl A. Retention in care and reasons for discontinuation of lifelong antiretroviral therapy in a cohort of Cameroonian pregnant and breastfeeding HIV-positive women initiating “Option B+” in the south west region. J Trop Med Int Health. (2017) 22(2):161–70. 10.1111/tmi.12816 27865052

[10] Kim MH Zhou A Mazenga A Ahmed S Markham C Zomba G Why did I stop? Barriers and facilitators to uptake and adherence to ART in Option B+ HIV care in Lilongwe, Malawi. PLoS One. (2016) 11(2):e0149527. 10.1371/journal.pone.0149527 26901563 PMC4762691

[11] Gouveia DC da Silva PA Pontes GA. Predictors of loss to follow-up among children registered in an HIV prevention mother-to-child transmission cohort study in Pernambuco, Brazil. J BMC Public Health. (2014) 14(1):1232. 10.1186/1471-2458-14-1232 PMC4289402 25430064

[12] Kyaw KWY Oo MM Kyaw NTT Phyo KH Aung TK Mya T Low mother-to-child HIV transmission rate but high loss-to-follow-up among mothers and babies in Mandalay, Myanmar; a cohort study. PLoS One. (2017) 12(9):e0184426. 10.1371/journal.pone.0184426 28886165 PMC5590939

[13] Dzangare J Takarinda KC Harries AD Tayler-Smith K Mhangara M Apollo TM HIV testing uptake and retention in care of HIV-infected pregnant and breastfeeding women initiated on “Option B+” in rural Zimbabwe. J Trop Med Int Health. (2016) 21(2):202–9. 10.1111/tmi.12637 26555353

[14] Haas AD Tenthani L Msukwa MT Tal K Jahn A Gadabu OJ Retention in care during the first 3 years of antiretroviral therapy for women in Malawi’s Option B+ program: an observational cohort study. J Lancet HIV. (2016) 3(4):e175–e82. 10.1016/S2352-3018(16)00008-4 PMC4904064 27036993

[15] Kiwanuka G Kiwanuka N Muneza F Nabirye J Oporia F Odikro MA Retention of HIV-infected pregnant and breastfeeding women on Option B+ in Gomba District, Uganda: a retrospective cohort study. J BMC Infect Dis. (2018) 18(1):533. 10.1186/s12879-018-3450-9 PMC6201534 30355356

[16] Kweyamba M Buregyeya E Kusiima J Kweyamba V Mukose AD. Loss to follow-up among HIV positive pregnant and lactating mothers on lifelong antiretroviral therapy for PMTCT in rural Uganda. J Adv Public Health. (2018). 10.1155/2018/7540587

[17] Llenas-García J Wikman-Jorgensen P Hobbins M Mussa MA Ehmer J Keiser O Retention in care of HIV-infected pregnant and lactating women starting ART under Option B+ in rural Mozambique. J Trop Med Int Health. (2016) 21(8):1003–12. 10.1111/tmi.12728 27208807

[18] Obai G Mubeezi R Makumbi F. Rate and associated factors of non-retention of mother-baby pairs in HIV care in the elimination of mother-to-child transmission program, Gulu-Uganda: a cohort study. J BMC Health Ser Res. (2017) 17(1):48. 10.1186/s12913-017-1998-5 PMC5241946 28100207

[19] Woelk GB Ndatimana D Behan S Mukaminega M Nyirabahizi E Hoffman HJ Retention of mothers and infants in the prevention of mother-to-child transmission of HIV programs is associated with individual and facility-level factors in Rwanda. J Int AIDS Soc. (2016) 19:20837. 10.7448/IAS.19.5.20837 27443268 PMC4956733

[20] Chan AK Kanike E Bedell R Mayuni I Manyera R Mlotha W Same-day HIV diagnosis and antiretroviral therapy initiation affect retention in Option B+ prevention of mother-to-child transmission services at antenatal care in Zomba District, Malawi. J Int AIDS Soc. (2016) 19(1):20672. 10.7448/IAS.19.1.20672 26976377 PMC4789547

[21] Ford D Muzambi M Nkhata MJ Abongomera G Joseph S Ndlovu M Implementation of antiretroviral therapy for life in pregnant/breastfeeding HIV+ women (Option B+) alongside rollout and changing guidelines for ART initiation in rural Zimbabwe: the lablite project experience. J Acquir Immune Defic Syndr. (2017) 74(5):508. 10.1097/QAI.0000000000001267 27984555 PMC5751886

[22] Haas AD Msukwa MT Egger M Tenthani L Tweya H Jahn A Adherence to antiretroviral therapy during and after pregnancy: cohort study on women receiving care in Malawi’s Option B+ program. J Clin Infect Dis. (2016) 63(9):1227–35. 10.1093/cid/ciw500 PMC5064160 27461920

[23] Landes M Sodhi S Matengeni A Meaney C van Lettow M Chan A Characteristics and outcomes of women initiating ART during pregnancy versus breastfeeding in Option B+ in Malawi. J BMC Public Health. (2016) 16(1):713. 10.1186/s12889-016-3380-7 PMC4973045 27487775

[24] Phillips T Thebus E Bekker LG Mcintyre J Abrams EJ Myer L. Disengagement of HIV-positive pregnant and postpartum women from antiretroviral therapy services: a cohort study. J Int AIDS Soc. (2014) 17(1):19242. 10.7448/IAS.17.1.19242 25301494 PMC4192834

[25] Tenthani L Haas AD Tweya H Jahn A van Oosterhout JJ Chimbwandira F Retention in care under universal antiretroviral therapy for HIV-infected pregnant and breastfeeding women (“Option B+”) in Malawi. J AIDS. (2014) 28(4):589. 10.1097/QAD.0000000000000143 PMC4009400 24468999

[26] Tweya H Gugsa S Hosseinipour M Speight C Ng'ambi W Bokosi M Understanding factors, outcomes, and reasons for loss to follow-up among women in Option B+ PMTCT program in Lilongwe, Malawi. J Trop Med Int Health. (2014) 19(11):1360–6. 10.1111/tmi.12369 25087778

[27] Mitiku I Arefayne M Mesfin Y Gizaw M. Factors associated with loss to follow-up among women in Option B+ PMTCT program in northeast Ethiopia: a retrospective cohort study. J Int AIDS Soc. (2016) 19(1):20662. 10.7448/IAS.19.1.20662 27005750 PMC4803835

[28] Tolossa T Kassa GM Chanie H Abajobir A Mulisa D. Incidence and predictors of lost to follow-up among women under Option B+ PMTCT program in western Ethiopia: a retrospective follow-up study. J BMC Res Notes. (2020) 13(1):18. 10.1186/s13104-019-4882-z PMC6947837 31910888

[29] Tesfaye DJ Hibistu DT Abebo TA Asfaw FT Lukas K Laelago T Option B plus antiretroviral therapy adherence and associated factors among HIV-positive pregnant women in southern Ethiopia. J BMC Pregnancy. (2019) 19(1):82. 10.1186/s12884-019-2228-4 PMC6394094 30819147

[30] Musomba R Mubiru F Nakalema S Mackline H Kalule I Kiragga AN Describing point of entry into care and being lost to program in a cohort of HIV-positive pregnant women in a large urban center in Uganda. J AIDS Res. (2017) 9(1):3527563. 10.1155/2017/3527563. PMC5392405 28469942

[31] Mukosha M Chiyesu G Vwalika B. Adherence to antiretroviral therapy among HIV infected pregnant women in public health sectors: a pilot of Chilenje level one Hospital Lusaka, Zambia. Pan Afr Med J. (2020) 35(49):49. 10.11604/pamj.2020.35.49.20078 32537054 PMC7250199

[32] Schnack A Rempis E Decker S Braun V Rubaihayo J Busingye P Prevention of mother-to-child transmission of HIV in Option B+ era: uptake and adherence during pregnancy in western Uganda. J AIDS Patient Care. (2016) 30(3):110–8. 10.1089/apc.2015.0318 27308804

[33] Hoffman RM Phiri K Parent J Grotts J Elashoff D Kawale P Factors associated with retention in Option B+ in Malawi: a case control study. J Int AIDS Soc. (2017) 20(1):21464. 10.7448/IAS.20.01.21464 28453243 PMC5515033

[34] Joseph J Gotora T Erlwanger AS Mushavi A Zizhou S Masuka N Impact of point-of-care CD4 testing on retention in care among HIV-positive pregnant and breastfeeding women in the context of Option B+ in Zimbabwe: a cluster randomized controlled trial. J Acquir Immune Defic Syndr. (2017) 75:S190–7. 10.1097/QAI.0000000000001341 28498189

[35] Langwenya N Phillips TK Brittain K Zerbe A Abrams EJ Myer L. Same-day antiretroviral therapy (ART) initiation in pregnancy is not associated with viral suppression or engagement in care: a cohort study. J Int AIDS Soc. (2018) 21(6):e25133. 10.1002/jia2.25133 29939483 PMC6016637

[36] Hoffman RM Phiri K Parent J Grotts J Elashoff D Kawale P Factors associated with retention in Option B+ in Malawi: a case-control study. J Int AIDS Soc. (2017) 20(1):21464. 10.7448/IAS.20.01.21464 28453243 PMC5515033

[37] Onoya D Sineke T Brennan AT Long L Fox MP. Timing of pregnancy, postpartum risk of virologic failure and loss to follow-up among HIV-positive women. J AIDS. (2017) 31(11):1593. 10.1097/QAD.0000000000001517 PMC5491237 28463877

[38] Demissie DB Erena MM Haile MT, editors. Assessment of loss to follow-up (LTFU) and associated factors among pregnant women initiated antiretroviral under option B+ in selected health facilities of west zone oromia, Ethiopia. J EC Gynaecol. (2019) 8:314–21.

[39] Central Statistical Agency. Federal democratic republic of Ethiopia central statistical agency population projection of Ethiopia for all regions at wereda level from 2014–2017. Available at: http://www.statsethiopia.gov.et/wp-content/uploads/2019/05/Population-Projection-At-Wereda-Level-from-2014-2017.pdf

[40] Ministry of Health (Ethiopia): national consolidated guidelines for comprehensive HIV prevention, care, and treatment (2018). Available at: https://www.childrenandaids.org/sites/default/files/2017-05/Ethiopia-Consolidated-ART-Guideline-2014.pdf

[41] Chi BH Yiannoutsos CT Westfall AO Newman JE Zhou J Cesar C Universal definition of loss to follow-up in HIV treatment programs: a statistical analysis of 111 facilities in Africa, Asia, and Latin America. PLoS Med. (2011) 8(10):e1001111. 10.1371/journal.pmed.1001111. 22039357 PMC3201937

[42] Cohen MS Chen YQ McCauley M Gamble T Hosseinipour MC Kumarasamy N Antiretroviral therapy for the prevention of HIV-1 transmission. N Engl J Med. (2016) 375(9):830–9. 10.1056/NEJMoa1600693 27424812 PMC5049503

[43] Gupta I Trivedi M Kandamuthan S. An analysis of recurrent costs of the free ART program of the government of India. J Growth Equity Environ Popul. (2006) 392.

[44] Grimsrud A Barnabas RV Ehrenkranz P Ford N. Evidence for scale-up: the differentiated care research agenda. J Int AIDS Soc. (2017) 20:22024. 10.7448/IAS.20.5.22024 28770588 PMC5577722

[45] Federal HIV/AIDS Prevention and Control Office. HIV prevention in Ethiopia national road map 2018–2020. Addis Ababa: Federal HIV/AIDS Prevention and Control Office (2018).

[46] Kim MH Ahmed S Hosseinipour MC Giordano TP Chiao EY Yu X Implementation and operational research: the impact of Option B+ on the antenatal PMTCT cascade in Lilongwe, Malawi. J Acquir Immune Defic Syndr. (2015) 68(5):e77. 10.1097/QAI.0000000000000517 25585302 PMC4359035

[47] Iroezi ND Mindry D Kawale P Chikowi G Jansen PA Hoffman RM. A qualitative analysis of the barriers and facilitators to receiving care in a prevention of mother-to-child program in nkhoma, Malawi. J Afr J Reprod Health. (2013) 17(4):118–29. PMCID: . 24689323 PMC4361063

[48] Megerso A Garoma S Tolosa Eticha TW Daba S Tarekegn M Habtamu Z. Predictors of loss to follow-up in antiretroviral treatment for adult patients in the Oromia region, Ethiopia. J HIV/AIDS. (2016) 8:83. PMCID: 10.2147/HIV.S98137 PMC4854271 27175095

[49] Aliyu A Adelekan B Andrew N Ekong E Dapiap S Murtala-Ibrahim F Predictors of loss to follow-up in art experienced patients in Nigeria: a 13-year review (2004–2017). BMC AIDS Res Ther. (2019) 16(1):30. 10.1186/s12981-019-0241-3 PMC6784330 31594539

